# Granulomatosis With Polyangiitis Presenting With Diffuse Alveolar Hemorrhage: A Systematic Review

**DOI:** 10.7759/cureus.29909

**Published:** 2022-10-04

**Authors:** Rafael C Da Silva, Prakash Adhikari

**Affiliations:** 1 School of Medicine, University Center for the Development of the Alto Vale of Itajai (UNIDAVI), Rio do Sul, BRA; 2 Internal Medicine, Piedmont Athens Regional Medical Center, Athens, USA; 3 Internal Medicine, Tower Health, Reading Hospital, Reading, USA

**Keywords:** c-anca/proteinase 3-positive granulomatosis with polyangiitis, anca-associated vasculitis, rituximab, systematic review, therapeutic plasmapheresis, respiratory failure, hemoptysis, proteinase 3 (pr3)-positive granulomatosis with polyangiitis (gpa), granulomatosis with polyangiitis (gpa), diffuse alveolar hemorrhage

## Abstract

Granulomatosis with polyangiitis (GPA) is a systemic disease that has variable clinical expression. GPA is the most common antineutrophilic cytoplasmic antibody* *(ANCA)-associated vasculitis (AAV). Diffuse alveolar hemorrhage (DAH) is one of the least common pulmonary manifestations in patients with GPA. DAH is clinically marked by hemoptysis, anemia, and diffuse alveolar infiltrates on imaging as well as hypoxemic respiratory failure. The diagnosis and treatment are challenging. Recommendations for ANCA-associated vasculitis, in general, are already established; however, there is a knowledge gap that addresses the association of GPA and DAH. The aim of this systematic review is to focus on the main clinical aspects and treatment of patients with GPA who present with DAH.

Thorough research of available literature was performed, including studies published in the last 10 years, following the Preferred Items for Systematic Review and Meta-Analysis (PRISMA) 2020 recommendations. The following databases were included: PubMed, Medline, Embase, ClinicalTrials.com, Google Scholar, and Prospero. The search terms included: [granulomatosis] AND [polyangiitis] AND [diffuse alveolar hemorrhage] OR [diffuse pulmonary hemorrhage] NOT [microscopic polyangiitis] NOT [eosinophilic granulomatosis]. NOS was used to assess the internal validity of the study in four domains, including selection, ascertainment, causality, and reporting. Our initial search identified 8989 studies. After excluding duplicated data and using our predetermined inclusion and exclusion criteria, we were able to retrieve 18 studies. Twelve out of 18 (67%) studies were case reports. Five were retrospective cohorts and one controlled trial. The average age of patients with GPA with DAH was 49.55 ± 17.54 years (18 - 76). Male individuals had a slight predominance (59%) in comparison to female individuals (41%). The hemoglobin level at the time of presentation was 8.86 mg/dL ± 1.43. The majority of patients (61.5%) reported hemoptysis. Renal involvement was present in 66.7%. Patients who required mechanical ventilation represented 61.5%. Plasmapheresis was used in 71.4%. Mortality was 20%, and gender was not associated with mortality (p = 0.822). Hemoptysis was not associated with the need for mechanical ventilation (p = 0.928). Renal impairment was not a predictor of mechanical ventilation (p = 0.207).

In summary, patients with GPA and DAH were severely ill, frequently had renal impairment upon admission, and frequently required mechanical ventilation. Steroids, rituximab, and cyclophosphamide are the first-line treatment, and plasmapheresis is still in use. Eventually, extracorporeal membrane oxygenation (ECMO) can be the salvage therapy. Randomized controlled clinical trials (RCTs) are needed to address the best therapeutic options for this population.

## Introduction and background

Granulomatosis with polyangiitis (GPA) is a systemic disease that can manifest with diffuse alveolar hemorrhage (DAH) in 5-15% of cases [1,2]. GPA is characterized by the formation of granulomas of the upper and lower respiratory tract secondary to necrotizing vasculitis of small and medium-sized vessels [3]. It has a higher prevalence in the age group of 45-60 years [3]. GPA is under the umbrella of antineutrophil cytoplasmic antibody (ANCA)-positive vasculitis (AAV), along with eosinophilic granulomatosis with polyangiitis (EGPA) and microscopic angiitis (MPA) [4]. DAH is a feared form of presentation of GPA. DAH is clinically marked by hemoptysis, anemia, diffuse alveolar infiltrates on imaging, and hypoxemic respiratory failure [5]. The presence of hemosiderin-laden macrophage on bronchoalveolar lavage (BAL) is considered diagnostic [6]. The average mortality ranges from 35% to 50% [2,7]. Hemoptysis is absent in about one-third of cases [8,9]. The underlying pathophysiology of DAH involves the disruption of the basement alveolar membrane with the subsequent accumulation of erythrocytes and posterior hemosiderin-laden macrophages within 48-72h [8]. On imaging, diffuse or focal ground glass opacities are characteristic. Alveolar opacities are common. In one study, 33% of DAH is immune-mediated, where GPA is the most common cause, followed by MPA and systemic lupus erythematosus (SLE) [7]. In the classic DAH triad of hemoptysis, new infiltrates on imaging, and anemia is found infrequently in 34% of cases [2]. DAH usually has nonspecific clinical and radiological findings. Frequently, BAL is necessary to accurately determine the diagnosis [1]. Bronchoscopy aims to rule out active bleeding of the large airways and active infection as a cause of DAH. BAL revealing more than 20% of the hemosiderin-laden macrophage is supportive of the diagnosis [1]. 

Review question

GPA with DAH is not a frequent presentation of pulmonary AAV. Recommendations for AAV, in general, are already established; however, there is a gap in knowledge addressing this association. The aim of this systematic review is to focus on the main clinical aspects and treatments of patients with GPA who present with DAH.

## Review

Methods

Search Methods

The protocol developed in this review followed the Preferred Items for Systematic Review and Meta-Analysis (PRISMA) 2020 standards [10]. A detailed investigation of the available literature was performed, including studies from 01/01/2012 to 05/01/2022. The following databases were included: PubMed/Medline, Embase, ClinicalTrials.com, Google Scholar, and Prospero. Furthermore, we searched the reference lists of included studies for any additional relevant publications. Corresponding authors of the selected studies were contacted to check for additional information or updates about their previously reported data. Search terms include [granulomatosis] AND [polyangiitis] AND [[diffuse alveolar hemorrhage] OR [diffuse pulmonary hemorrhage]] NOT [microscopic polyangiitis] NOT [eosinophilic granulomatosis].

Eligibility Criteria

The studies included in this research met the following inclusion criteria: Studies reporting patients with GPA AND DAH (Appendix A for diagnosis criteria); Studies published from 01/01/2012 to 05/01/2022; Studies reporting patients older than 18 years of age; Case reports, case series, retrospective and prospective cohorts, and clinical trials.

On the other hand, studies were excluded based on the following conditions: A diagnosis of vasculitis other than granulomatosis with polyangiitis; Studies reporting patients under the age of 18; Studies published in languages other than English; Letters to the editors, book chapters, conferences, abstracts, and editorials

The selection process was performed by both authors independently. After the initial search, repeated articles were excluded. After the initial selection, the titles and abstracts were reviewed for further consideration by both authors.

Data Extraction

The data aimed to be collected included demographics, namely, age and gender. Clinical parameters included the presence of hemoptysis, hemoglobin levels (mg/dL), and renal injury (yes or no) at the time of presentation. In addition, the need for invasive mechanical ventilation was collected. Finally, the core pharmacological treatment used includes steroids alone or in combination with cyclophosphamide, rituximab, or a combination of them. Additional treatments, such as plasmapheresis and extracorporeal membrane oxygenation (ECMO), were also extracted. The primary outcome sought was mortality, reported as hospital discharge or death. The corresponding authors of the selected studies were contacted by email to retrieve any missing data from the body of the article. Missing data were analyzed and treated using statistical software. Categorical variables were reported as percentages. For continuous variables, mean ± SD was used. For the comparison of binomial categorical variables, the chi-square test was used. A p-value of less than 0.05 was considered statistically significant. Statistic data analysis was carried out using GNU PSPP version 1.4.1 software (https://www.gnu.org/software/pspp/).

Assessment of Risk of Bias

Population selection, comparability, and ascertainment of exposure/outcome were assessed using the Newcastle Ottawa Scale (NOS) on the outcome level across the studies or the NOS modified scale for case report/case series, as proposed by Murad MH, Sultan S, Haffar S, et al. [11]. NOS was used to assess the internal validity of the study in four domains including selection, ascertainment, causality, and reporting (Appendix B).

Results

**Figure 1:**
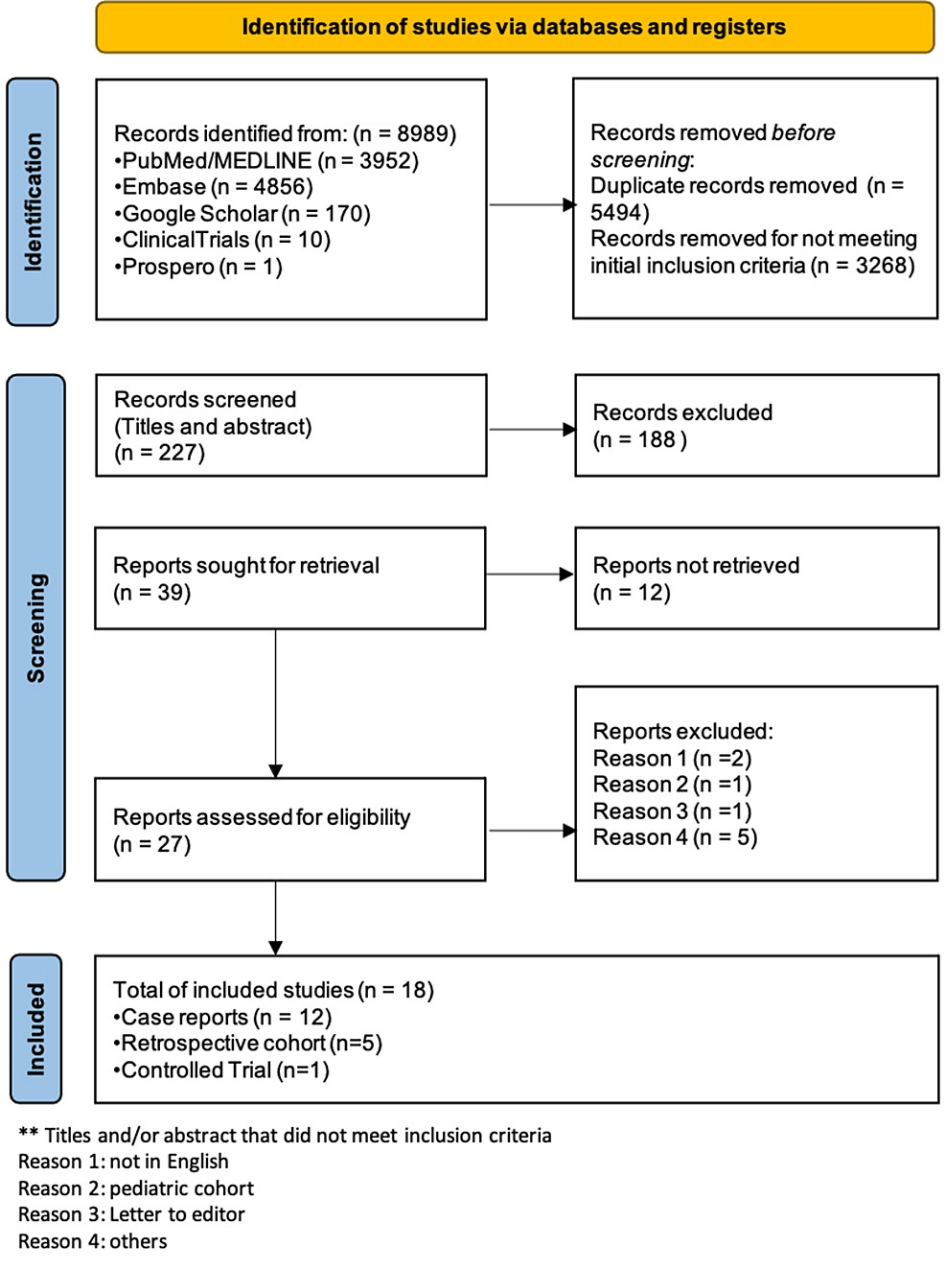
PRISMA 2020 flow diagram of the research process PRISMA: Preferred Reporting Items for Systematic Reviews and Meta-Analyses

The summary of the research process was plotted as suggested by the PRISMA 2020 flow diagram (Figure 1). Our initial search identified 8989 studies from 01/01/2012 to 05/01/2022. After excluding duplicated data and using our predetermined inclusion and exclusion criteria, we were able to retrieve 18 studies. Interestingly, 12 of 18 (67%) studies were case reports. Five were retrospective cohorts and one was a controlled trial (Figure 1).

Table 1 presents a summary of the characteristics of the included case reports.

**Table 1:** Summary of the studies with case reports of GPA and DAH 1: Hemoptysis, 2: hemoglobin, 3: renal involvement, 4: invasive mechanical ventilation GPA: granulomatosis with polyangiitis; DAH: diffuse alveolar hemorrhage

Study	age	sex	hemo^1^	hbg^2^	renal^3^	IMV^4^	outcome
Moreno-González et al.(2014) [12]	42	M	Y	9.2	Y	N	discharged
Vanoli et al. (2017) [13]	33	M	Y	9	Y	Y	discharged
Ning et al. (2018) [14]	70	F	N	5	N	Y	discharged
Watanabe et al. (2018) [15]	65	M	Y	11.3	Y	N	discharged
Collada et al. (2019) [16]	72	F	Y	8.6	N	N	discharged
Sattar et al. (2019) [17]	60	F	Y	5.8	Y	Y	discharged
Yin et al. (2020) [18]	25	F	N	10.4	Y	Y	discharged
	18	F	Y	n/a	Y	Y	discharged
Li et al. (2021) [19]	52	M	N	10.8	N	N	discharged
Wang et al. (2021) [20]	56	M	N	8.6	Y	Y	discharged
Jin et al (2022) [21]	76	M	Y	7.7	Y	Y	discharged
Madan et al. (2022) [4]	34	F	N	n/a	N	N	discharged
Pereira et al. (2022) [22]	25	M	Y	11.1	N	Y	discharged

Table 2 presents the main characteristics of the retrospective studies. The risk of bias assessment summary for each study can be found in Appendix C.

**Table 2:** Total and percentage of patients with GPA and GPA with DAH in selected retrospective and RCT studies are presented GPA: granulomatosis with polyangiitis; DAH: diffuse alveolar hemorrhage; RCT: randomized control trial

Study	total of patients	GPA with DHA	%
Gómez-Gómez et al. (2014) [23]	14	2	14%
Quadrelli et al. (2017) [6]	39	14	35%
Cortazar et al. (2018) [24]	129	20	16%
Bhushan et al. (2021) [7]	88	9	10%
Loftis et al. (2022) [5]	19	7	36%
Walsh et al. [25]	704	62	8,8%

From the data collection available in the case reports, the average age of patients with GPA with DAH was 49.55 ± 17.54 years (18-76). Male individuals had a slight predominance (59%) in comparison to female individuals (41%). The hemoglobin level at the time of presentation was 8.86 mg/dL ± 1.43. Most patients (61.5%) reported hemoptysis at presentation. Renal involvement was present in 66.7% of the patients. In the same way, patients requiring mechanical ventilation were reported as higher at 61.5%. In terms of treatment, plasmapheresis was used in 71.4%, rituximab in 25%, and ECMO in 30.8% when using case report data. The mortality of 20%. Gender was not associated with mortality (p = 0.822). Hemoptysis was not associated with the need for mechanical ventilation (p = 0.928). Renal impairment was not a predictor of mechanical ventilation (p = 0.207).

Discussion

In this systematic review, we focused on one ANCA-associated vasculitis (AAV), namely, GPA, so one can delineate an overall clinical picture of patients with the diagnosis of GPA presenting with DAH. Eighteen studies were retrieved, 12 (67%) case reports, five (28%) retrospective cohorts, and one (5%) randomized control trial (RCT). The paucity of randomized controlled trials and prospective studies is likely due to the rarity of DAH as a form of presentation of GPA. Furthermore, the study focused only on GPA as an immune-mediated cause of vasculitis, excluding MPA, EGPA, SLE, and anti-glomerular membrane basement disease, contributing to the low sample and the need to include case reports in this analysis. It was interesting that all the patients included in the case reports were discharged. No death was reported. We believe this is likely associated with a bias of publication, considering the low chance of publishing case reports whose outcomes include death or unsuccessful treatment.

The pathophysiology of the DAH involves the impairment of the basement membrane of the alveoli leading to permeability to erythrocytes, with an eventual accumulation of hemosiderin-laden macrophages within 48 to 72h [7]. GPA has variable clinical expression, including pulmonary manifestation. Even less common is the DAH presentation. It usually represents a diagnostic and therapeutic challenge. Differentials include pulmonary edema, bacterial or viral pneumonia, and other forms of autoimmune vasculitis such as anti-glomerular antibodies basement membrane disease, MPA, EGPA, and SLE vasculitis. Recognition must be prompt because treatment requires aggressive immunosuppression and other therapies such as plasmapheresis or even ECMO.

Our systematic review found that patients with GPA presenting with DAH are 49.55 ± 17.54 years on average. They are male predominantly (59%). Low hemoglobin level is a constant clinical feature, and almost all patients have anemia at presentation. The majority of them (61.5%) report hemoptysis on presentation, which is in accordance with previous literature [6,8,26]. However, the absence of it does not exclude the diagnosis, considering that hemoptysis has a low sensitivity to the diagnosis of DAH [1,8,27]. Renal involvement and the need for mechanical ventilation are present in two-thirds of the cases. We believe that the high frequency of renal impairment is associated with the high level of disease activity and circulating antibodies in cases of DAH.

In terms of treatment, plasmapheresis was used in 71%. This was in discordance with Walsh et al. in their RCT, which showed that plasmapheresis did not reduce the incidence of death or end-stage renal disease (ESRD) [25]. In addition, the recommendation by the 2021 American College of Rheumatology/Vasculitis Foundation Guidelines supports this finding. Accordingly, in patients with active, severe GPA with alveolar hemorrhage, the guidelines conditionally recommend against adding plasma exchange to remission induction therapies [28]. However, plasma exchange may be considered for certain patients with active forms of glomerulonephritis or those who are critically ill and whose disease is not responding to recommended remission induction therapies (i.e., plasma exchange as “salvage” or “rescue” therapy).

High doses of steroids, cyclophosphamide, and rituximab were frequently used. We did notice more frequent use of rituximab over the years. This is probably an effect of the RAVE trial when rituximab was shown to be non-inferior to cyclophosphamide and could be even superior in relapsing disease [29]. ECMO was used as rescue therapy in some patients. ECMO is an alternative for patients who failed conventional mechanical ventilation to treat DAH in patients with ANCA-associated vasculitis [20].

Summary of Retrospective Cohorts

1. In the study of Gómez-Gómez et al., a cohort of 14 patients was studied [23]. Two patients of 14 (14%) had DAH. In both patients, chronic renal disease was present. The diagnosis was confirmed by CT of the thorax showing diffuse ground glass opacity (GGO) and positive BAL. One of the patients with DAH required invasive mechanical ventilation (IMV).

2. Quadrelli et al. in their cohort reported 14 patients with DAH of 39 (35%) [6]. In 76% of the patients, hemoptysis was present. The typical imaging was bilateral GGO on a CT scan of the thorax. Five patients received rituximab as treatment in addition to steroids and cyclophosphamide. One-third required mechanical ventilation and one-third had renal failure upon admission. In this cohort, plasmapheresis was required for 40%. The mortality rate was 33%.

3. In the Cortazar et al. study, 129 patients were enrolled in a retrospective cohort of 10 years to evaluate three drug-standardized treatment regimens, including rituximab, cyclophosphamide, and low-dose steroid [24]. Patients identified with severe forms of GPA with DAH or rapidly progressive glomerulonephritis received pulse methylprednisolone and plasmapheresis in the standard regimen. In that series, 20 (16%) out of 129 presented DAH associated with GPA. Only four deaths were reported and only one patient presented with hemoptysis, different from the rest of the literature. In this series, DAH and the use of plasmapheresis are associated with an increased risk of serious infection.

4. Bhushan et al. reported a retrospective cohort of 12 years of 88 cases of DAH of immune and non-immune causes [7]. In this study, the minority of cases (37%) of DAH were thought to be immune-mediated. Among those, GPA was the most common, with nine patients (10%) reported. Like in our study, all cohorts presented anemia at the time of diagnosis. Plasmapheresis was used in 78% of cases. Almost two-thirds required invasive mechanical ventilation and nearly all cases had renal impairment at the diagnosis. The mortality rate was 15%.

5. In the Loftis et al. study, 19 patients with a diagnosis of DAH associated with ANCA vasculitis were analyzed [5]. Interesting, GPA was less frequent in this cohort, with only seven patients (37%). In this study, the presence of hemoptysis was higher, at 84%. Mechanical ventilation was required in 47% and renal involvement was 95% present at the time of diagnosis. Anemia, as in the other studies, was present in all patients. Plasmapheresis was used in 42% of patients. Mortality was 36%. Rituximab was used in 79%, which is consistent with the noninferiority RAVE trial of rituximab compared to cyclophosphamide [29].

6. Walsh et al. conducted a randomized controlled trial in 2020 [25]. The aim was to determine the effect of plasmapheresis and two different oral steroids (standard and low dose) in the treatment of severe forms of AAV. Both GPA and MPA patients were recruited. Severe DAH was found in 8.8% of patients. The need for dialysis ranged from 18% to 21%. In this study, the addition of plasma exchange to the standard therapy did not confer benefits to patients with severe AAV in terms of mortality or end-stage kidney disease. On the other hand, it did show that a reduced-dose regimen of oral glucocorticoids was non-inferior to a standard-dose regimen.

Strengths

One of the main strengths of this systematic review is the focus on diffuse alveolar hemorrhage related to patients with GPA, one of the most common AAVs. It differs from other studies, in which other AAV and other auto-immune diseases are included in the analysis such as MPA, EGPA, and SLE.

Limitations

Due to the scarcity of data and the low prevalence, it was necessary to add case reports in this review, which can potentially compromise external validity. In addition, GPA with DAH is a non-common manifestation of AAV. Gathering enough patients to design prospective RCTs is difficult, which compromises statistical and clinical significance.

Future Considerations

It is still not clear what are the risk factors for the development of DAH in patients with GPA. Are early detection and treatment able to prevent further clinical deterioration? Furthermore, is there any long-term complication of DAH such as lung fibrosis? We do believe that randomized controlled trials addressing those questions will be welcomed, however, due to the not-so-common presentation, this could pose a difficulty as previously discussed.

## Conclusions

Granulomatosis with polyangiitis (GPA) is a systemic disease that has variable clinical expression, including pulmonary manifestations. Even less common is DAH. It usually represents a diagnostic and therapeutic challenge. Differentials include pulmonary edema, bacterial or viral pneumonia, and other forms of autoimmune vasculitis. Patients with GPA and DAH are predominantly men of 49 years of age on average, with anemia and often hemoptysis. They are severely ill, with renal impairment upon admission, and not rarely require mechanical ventilation. Steroids, rituximab, and cyclophosphamide are the base of therapy, and plasmapheresis is still in place. Eventually, ECMO can be the salvage therapy. RCTs are needed to address the best therapeutic options for this population.

## Figures and Tables

**Figure 2:**
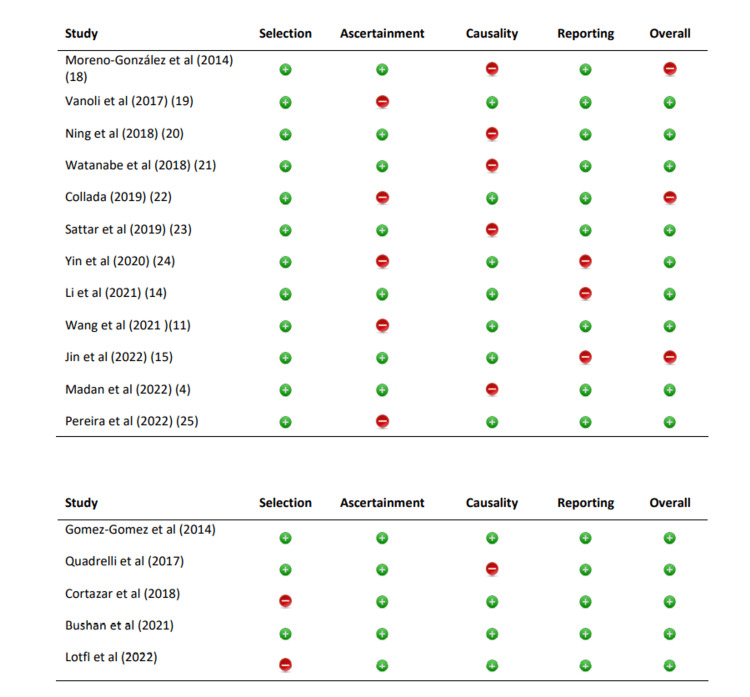
The risk of bias assessment summary for each study

## Appendices

Appendix A

Diagnostic criteria for GPA

1.     New or previous relapsing clinical diagnosis of granulomatosis with polyangiitis consistent with the Chapel-Hill consensus definitions

Diagnostic criteria for DAH

Pulmonary hemorrhage due to active vasculitis is defined by the following:

1.     A compatible chest x-ray or CT scan (diffuse pulmonary infiltrates)

AND

2.     The absence of an alternative explanation for all pulmonary infiltrates (i.e. volume overload or pulmonary infection)

AND

3. At least one of the following:

a. Evidence of alveolar hemorrhage on bronchoscopy or increasingly bloody returns with bronchoalveolar lavage

b. Observed hemoptysis

c. Unexplained anemia (<10 g/dL) or documented drop in hemoglobin (>1 g/dL) from less than 10g/dl

d. An increased diffusing capacity of carbon dioxide

Appendix B

**Table 3:** Newcastle Ottawa Scale (NOS) for evaluating the methodological quality of case reports and case series

Domains	Leading explanatory questions
Selection	1. Does the patient(s) represent(s) the whole experience of the investigator (center) or is the selection method unclear to the extent that other patients with a similar presentation may not have been reported?
Ascertainment	2. Was the exposure adequately ascertained? 3. Was the outcome adequately ascertained?
Causality	4. Were other alternative causes that may explain the observation ruled out? 5. Was there a challenge/rechallenge phenomenon? 6. Was there a dose-response effect? 7. Was follow-up long enough for outcomes to occur?
Reporting	8. Is the case(s) described with sufficient detail to allow other investigators to replicate the research or to allow practitioners to make inferences related to their own practice?

Appendix C
